# Aortic arch approach using clampless anastomosis for high-flow microaxial pump: An alternative in challenging anatomy

**DOI:** 10.1016/j.xjtc.2025.102182

**Published:** 2025-12-12

**Authors:** Yoshinori Nakahara, Tomohiro Iwakura, Akira Marui, Kohei Sumi, Ryogen Yun, Makoto Ono

**Affiliations:** Department of Cardiovascular Surgery, Sakakibara Heart Institute, Tokyo, Japan

**Figure:**
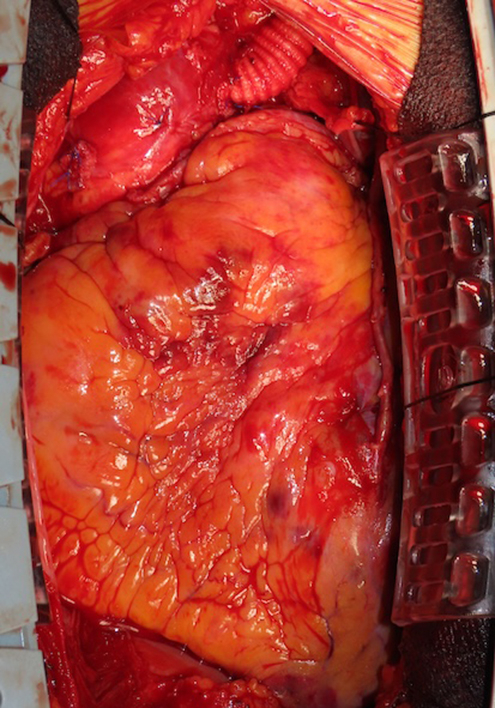
Aortic arch graft for Impella 5.5 insertion using clampless anastomosis.

**Central Message**

Aortic arch approach using clampless anastomosis is a feasible alternative for Impella 5.5 insertion when conventional approaches are unsuitable.

Impella 5.5 (Abiomed, Inc) is a high-flow microaxial pump, a mechanical circulatory support device capable of delivering flows exceeding 5.0 L/min for the treatment of cardiogenic shock. Successful Impella 5.5 insertion requires specific anatomical conditions as recommended by the manufacturer: subclavian arteries measuring at least 7 mm in diameter for the transaxillary approach or a valve-to-graft distance exceeding 70 mm for the direct aortic approach.^1^

The Enclose II (Peters Surgical) creates a bloodless anastomotic field by sandwiching the aortic wall between upper and lower jaws without clamping.^2^ We report an aortic arch graft approach using the Enclose II for Impella 5.5 insertion when conventional approaches are anatomically unsuitable.

## Methods

This study was approved by the institutional review board of Sakakibara Heart Institute on October 21, 2025 (approval number: 25-048). Written informed consent was obtained from the patient for publication of this case report and accompanying images.

## Case Presentation

**Figure 1:**
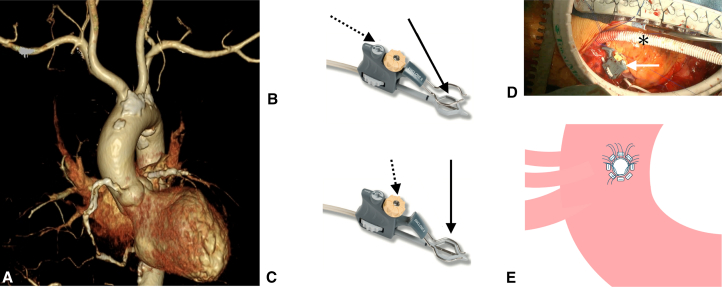
Surgical technique for aortic arch graft anastomosis. A, Computed tomography showing calcified aorta and small subclavian arteries. B, Opening the lower jaw membrane. Turn the switch (*dashed arrow*) with the actuator tool or lower knob using fingers to open the lower jaw membrane (*solid arrow*). C, Creating a blood-tight seal. Turn the upper knob (*dashed arrow*) with the actuator tool or fingers to lower the position of the upper jaw (*solid arrow*), enabling a blood-tight seal and barrier. D, Aortic arch graft anastomosis using the Enclose II device. The Enclose II device (*white arrow*) is positioned on the aortic arch, creating a bloodless anastomotic field without aortic crossclamping. A 10-mm prosthetic graft (*asterisk*) is anastomosed end-to-side using interrupted 4-0 polypropylene sutures with felt pledgets (*left*: cranial; *right*: caudal). E, Schematic illustration of the anastomosis site on the aortic arch. Eight interrupted 4-0 polypropylene mattress sutures with felt pledgets are placed between the left common carotid artery and the left subclavian artery.

A 64-year-old man (body surface area 1.80 m^2^) with ischemic cardiomyopathy (ejection fraction 27.3%, European System for Cardiac Operative Risk Evaluation II 5.85%) scheduled for cardiac surgery required mechanical circulatory support. Enhanced computed tomography revealed bilateral subclavian arteries <6 mm in diameter and partial ascending aortic calcification (Figure 1, *A*). The distance from the aortic valve to an available ascending aortic site was 50 mm. The aortic arch between the left common carotid and left subclavian arteries showed no calcification, making it suitable for graft insertion.

### Surgical Technique

After median sternotomy and graft harvest, the Enclose II device was positioned with the upper jaw on the adventitial surface and lower jaw on the intimal surface (Video 1). The lower jaw was expanded (Figure 1, *B*) and the upper jaw compressed the aortic wall (Figure 1, *C*), creating a bloodless field without interrupting perfusion.

Within the 15-mm footprint, an arteriotomy was created using a beaver blade and 4-mm punch. A 10-mm graft was anastomosed end-to-side using 8 interrupted 4-0 polypropylene mattress sutures with felt pledgets (Figure 1, *D* and *E*, Video 2). After anastomosis, the device was removed and hemostasis confirmed.

Approximately 3 cm distal to the anastomosis, a T-configuration was created by side-to-side anastomosis with another 10-mm graft (Video 2). This side branch served as arterial inflow for cardiopulmonary bypass.

**Figure 2:**
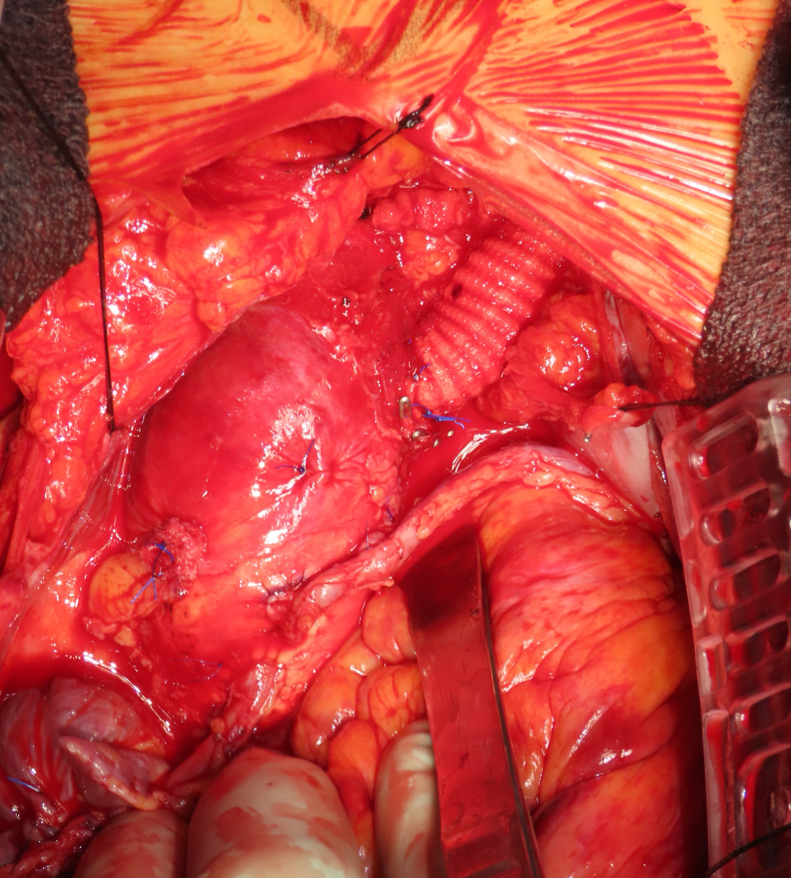
Impella through arch graft with surgical clips.

After surgical ventricular restoration and coronary bypass grafting with 6 grafts, the main graft was tunneled subcutaneously to the left supraclavicular region for subsequent Impella insertion. Under transesophageal echocardiography guidance, the Impella 5.5 catheter was advanced through the main graft across the aortic valve into the left ventricle with outlet 3 to 4 cm above the aortic valve (Video 3).^3^ The anastomosis site was marked with surgical clips (Figure 2). The valve-to-graft distance was 90 mm, exceeding the 70-mm minimum. The patient was separated from bypass and the side-branch graft was ligated.

Postoperatively, the Impella 5.5 provided excellent support with flows of 4.0 to 4.5 L/min. The patient was weaned from the Impella on postoperative day 7. Without resternotomy, after the Impella was removed from the prosthetic graft, the remaining graft was shortened as much as possible, the stump was oversewn to close it, then it was buried subcutaneously before the skin was closed.

## Discussion

This case demonstrates a feasible alternative when conventional approaches are contraindicated. The transaxillary approach remains preferred but requires vessel caliber ≥7 mm.^4^ Alternative strategies include the suprasternal approach,^5^ brachiocephalic artery access,^4^ and the direct aortic approach.^3^ The conventional direct aortic approach requires a valve-to-graft distance ≥70 mm, which is often unattainable in small patients. The aortic arch approach overcomes both constraints.

The aortic arch approach using the Enclose device avoids manipulation of calcified tissue, minimizing embolic risk. In addition, the 15-mm upper jaw provides adequate space for secure 10-mm graft anastomosis. Interrupted mattress sutures with felt pledgets prevent aortic tissue tear and provide superior mechanical strength during Impella catheter insertion and removal.

For patients undergoing concomitant cardiac surgery, this approach can offer dual functionality. By creating a T-configuration anastomosis to the arch graft, the side branch can easily serve as arterial inflow for cardiopulmonary bypass while the main graft provides Impella access, eliminating separate aortic cannulation. Finally, device explantation does not require resternotomy; the graft is accessed through the original supraclavicular incision.

Important technical considerations include the requirement for a disease-free aortic arch segment, operator expertise with the Enclose device, and mandatory intraoperative transesophageal echocardiography guidance.^3^ Subcutaneous graft tunneling may theoretically increase infection risk, though no infectious complications occurred in this case.

## Conclusions

Aortic arch approach using the Enclose II clampless device is a feasible alternative for Impella 5.5 insertion when subclavian arteries are inadequate and the ascending aorta is unsuitable.

## Conflict of Interest Statement

The authors reported no conflicts of interest.

The *Journal* policy requires editors and reviewers to disclose conflicts of interest and to decline handling or reviewing manuscripts for which they may have a conflict of interest. The editors and reviewers of this article have no conflicts of interest.

## Supplementary Data

Video 1 Enclose II device deployment creating bloodless field. Video available at: https://www.jtcvs.org/article/S2666-2507(25)00563-2/fulltext.

Video 2 Aortic arch graft anastomosis, device removal, and T-configuration side branch creation. Video available at: https://www.jtcvs.org/article/S2666-2507(25)00563-2/fulltext.

Video 3 Impella 5.5 insertion through arch graft under transesophageal echocardiography guidance. Video available at: https://www.jtcvs.org/article/S2666-2507(25)00563-2/fulltext.
