# Supplementary figures and images for: Aortic arch approach using clampless anastomosis for high-flow microaxial pump: An alternative in challenging anatomy
Source: JTCVS Tech. 2025 Dec 12;35:102182. doi: 10.1016/j.xjtc.2025.102182 (PMC12881765; doi:10.1016/j.xjtc.2025.102182)

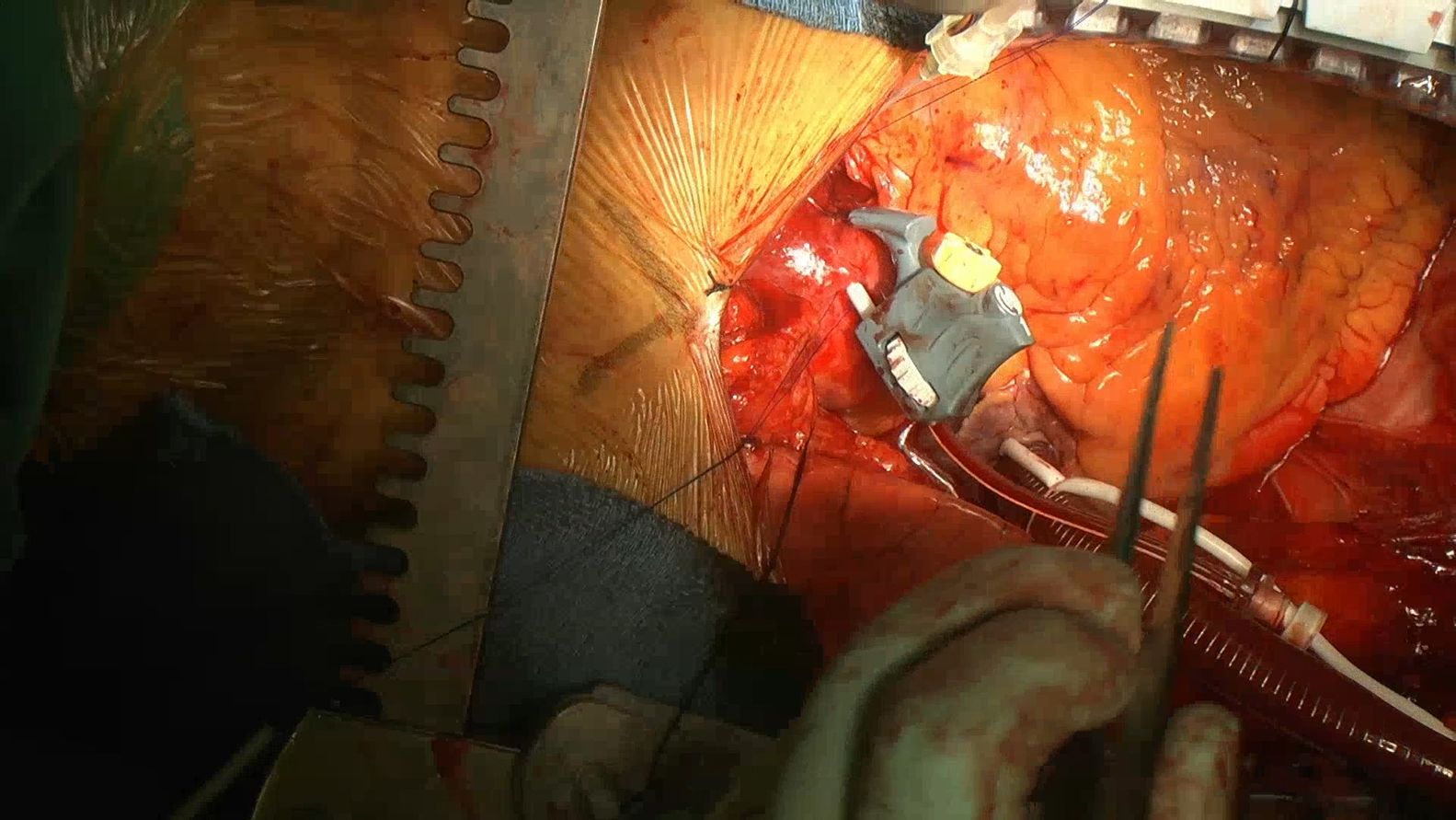

Supplement: Video 1 — Enclose II device deployment creating bloodless field. Video available at: https://www.jtcvs.org/article/S2666-2507(25)00563-2/fulltext. [file fx2.jpg]

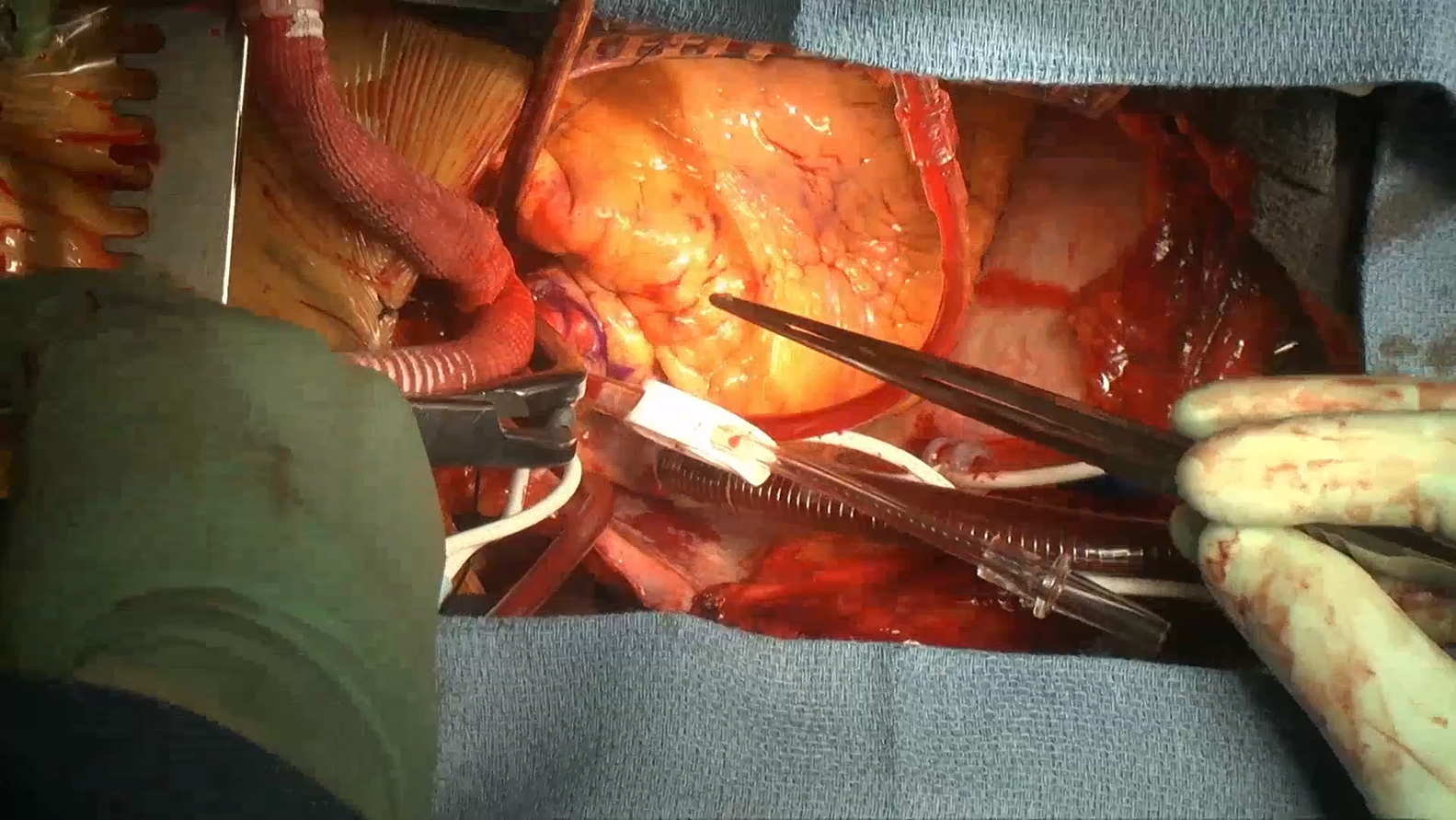

Supplement: Video 2 — Aortic arch graft anastomosis, device removal, and T-configuration side branch creation. Video available at: https://www.jtcvs.org/article/S2666-2507(25)00563-2/fulltext. [file fx3.jpg]

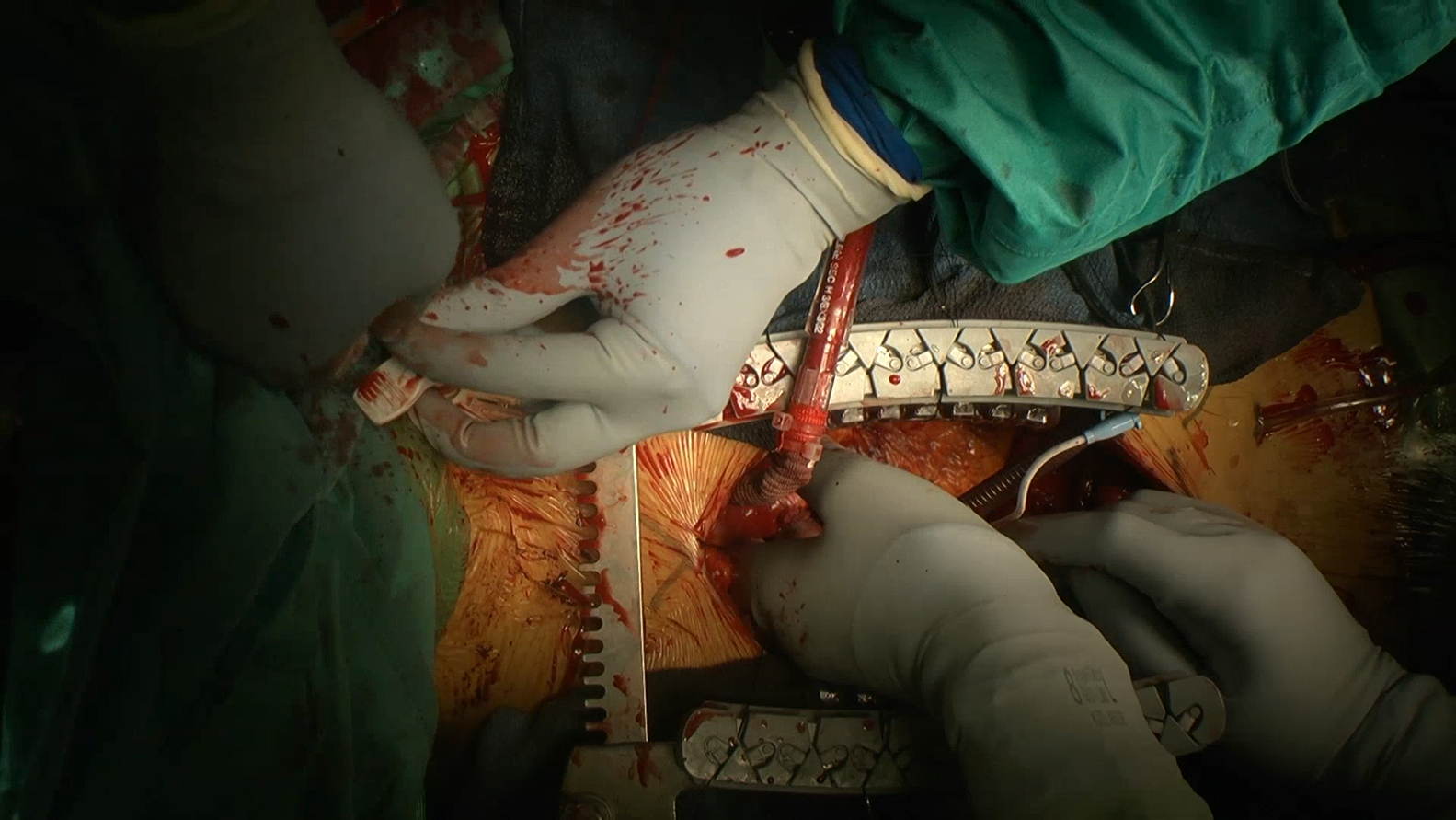

Supplement: Video 3 — Impella 5.5 insertion through arch graft under transesophageal echocardiography guidance. Video available at: https://www.jtcvs.org/article/S2666-2507(25)00563-2/fulltext. [file fx4.jpg]
